# Dislocation of the Shoulder

**Published:** 1883-04-20

**Authors:** 


### DISLOCATION OF THE SHOULDER.

[A Contribution in Support of Kelley's Method of Reduction.]

Mrs. ——, widow, charwoman, aged about 40, presented herself at the out-patient department, Toronto General Hospital, saying that some thirteen days previously, while returning from work in the evening, she had slipped and fallen and hurt her shoulder. At the time of examination there was no swelling nor bruising, but marked flattening of the left shoulder existed, with some prominence and marked tenderness beneath the coracoid process. The underhand motions of the arm were pretty free, and her hand could be placed on the opposite shoulder, and even on the top of the head with considerable facility. The surgeon's fingers could be readily made to explore the glenoid cavity, as the patient was thin, and the lower portion of the head could be felt in the axilla upon elevation and rotation of the arm. The free movement of the arm and the capacity to place the hand on the opposite shoulder and on the head were certainly unusual, but Dugas's test and the other evidences of luxation mentioned were too positive to be mistaken. There was no shortening of the limb. Reduction was first attempted by the method recommended by Kocher, at the late meeting of the International Congress in London. Kocher's method is as follows:

The patient is seated, with the surgeon on his left hand. The elbow-joint is first to be flexed to a right angle, and the joint firmly pressed against the side of the chest; then, while holding the elbow in contact with the body, the arm is to be slowly, gently and steadily rotated out until firm resistance is encountered; then, maintaining this rotation, the arm is to be raised forwards and a little in, and lastly, to be rotated in, and the hand brought towards the opposite shoulder. No anæsthetic is needed, and Ceppi says the method is especially valuable in old dislocations.

This was repeated a second time without avail. Kelley's method (which consists in placing the patient on a firm table of convenient height, lying upon the back so as to fix the scapula by the weight of the body, with the side of the luxation drawn well to the edge, while the surgeon extends the arm to a right angle with the body, and then places one of his hips against the patient's side well up in the axilla, and draws the extended arm around his pelvis, holding the hand firmly fixed upon his illium, after which position is secured, he suddenly or slowly rotates his body on its vertical axis until his back lies parallel with the patient's side), was then tried, and with a minimum of effort was at once crowned with complete success, some tearing of tissue being plainly heard, and the head of the bone returning to its socket with an audible and sensible snap.—*Canadian Journal.*

---

THERE is eminent medical authority for the statement that unripe or very old potatoes contain a certain quantity of solanine. This may produce serious results, if the potatoes are boiled with their skins on, and if they are eaten in large quantities.—*Ex.*

